# Metabolic Syndrome in Overweight Or Obese Children and Adolescents Based on Own Material

**DOI:** 10.34763/devperiodmed.20182204.351357

**Published:** 2019-01-14

**Authors:** Katarzyna Szabelska-Zakrzewska, Alina Durko, Anna Socha-Banasiak, Małgorzata Majewska, Michał Kolejwa, Joanna Kazanek-Zasada, Elżbieta Czkwianianc

**Affiliations:** 1 Department of Gastroenterology, Allergology and Pediatrics, Polish Mother’s Memorial Hospital – Research Institute, Lodz, Poland

**Keywords:** metabolic syndrome, obesity, lipid disorders, children, diabetes, zespół metaboliczny, otyłość, zaburzenia przemiany lipidowej, dzieci, cukrzyca

## Abstract

**Aim:**

*To assess the prevalence of metabolic syndrome (MS) components in overweight or obese children and adolescents, as well as analyze the risk factors of its occurrence*.

**Material and methods:**

*The study was conducted in a group of 70 children and adolescents aged 5-18 hospitalized in the Department of Gastroenterology, Allergology and Pediatrics, Polish Mother’s Memorial Hospital – Research Institute in Lodz (Poland) based on the results of medical histories, physical examination, biochemical investigation, and calculation of the Homeostatic Model Assessment of Insulin Resistance (HOMA-IR) index*.

**Results:**

*MS was diagnosed in 14 children (20%). The most common abnormalities besides obesity included: decreased High Density Lipoprotein Cholesterol (HDL-C) levels (n=13, 92.9%), increased triglycerides (TG) concentrations (n=10, 71.4%) and arterial hypertension (n=10, 71,4%). Among all the children, insulin resistance was diagnosed in 29 subjects (41.4%). The results of univariate logistic regression showed that the occurrence of lipid disorders, obesity, hypertension and diabetes in their parents, as well as the duration of pregnancy, birth weight, or breastfeeding were not associated with the risk of MS development in the subjects (p>0.05). However, in the study group, 92.9% of subjects had one or more particular risk factor for MS development*.

**Conclusions:**

*Besides visceral obesity, lipid disorders were the most frequently observed components of MS in the subjects analyzed, which may have prognostic significance. The occurrence of one or more MS risk factors in almost all of the children studied indicates the increased risk of cardiovascular diseases in the studied group in the future*.

## Streszczenie

**Cel:**

*Ocena częstości występowania poszczególnych składowych zespołu metabolicznego u dzieci i młodzieży z otyłością lub nadwagą oraz analiza czynników ryzyka jego wystąpienia*.

**Materiał i metody:**

*Badanie zostało przeprowadzone w grupie 70 dzieci i młodzieży w wieku 5-18 lat, hospitalizowanych w Klinice Gastroenterologii, Alergologii i Pediatrii Instytutu Centrum Zdrowia Matki Polki w Łodzi. Przeprowadzono wywiady medyczne, badanie przedmiotowe, badania biochemiczne oraz obliczono wskaźnik HOMA-IR (ang. Homeostatic Model Assessment of Insulin Resistance)*.

**Wyniki:**

*Zespół metaboliczny rozpoznano u 14 dzieci (20%). Najczęstszymi nieprawidłowościami oprócz otyłości były: obniżenie stężenia frakcji HDL (ang. High Density Lipoprotein Cholesterol) (n=13, 92,9%), wzrost stężenia triglicerydów (n=10, 71,4%) oraz nadciśnienie tętnicze (n=10, 71,4%). Insulinooporność stwierdzono u 29 dzieci (41,4%). Wyniki jednoczynnikowej regresji logistycznej wykazały, że występowanie u rodziców badanych dzieci zaburzeń lipidowych, otyłości, nadciśnienia tętniczego, cukrzycy jak również długość trwania ciąży, urodzeniowa masa ciała, karmienie naturalne nie miały wpływu na ryzyko wystąpienia zespołu metabolicznego (p>0,05). Jednak 92,9% badanych miało jeden lub więcej czynników ryzyka rozwoju zespołu metabolicznego*.

**Wnioski:**

*Z wszystkich kryteriów rozpoznania zespołu metabolicznego w badanej grupie najczęściej oprócz otyłości trzewnej były stwierdzane zaburzenia przemiany lipidowej, co może mieć znaczenie prognostyczne. Stwierdzenie u prawie wszystkich badanych jednego lub więcej czynników ryzyka rozwoju zespołu metabolicznego wskazuje na zwiększone ryzyko występowania u nich w przyszłości chorób układu sercowo-naczyniowego*.

## Introduction

Metabolic syndrome (MS) comprises a group of metabolic and hemodynamic disorders including: visceral fat obesity, disturbances of carbohydrate and lipid metabolism, as well as hypertension. The presence of MS 5-fold increases the risk of diabetes and is an element that should be considered in the assessment of long-term risk of developing cardiovascular diseases, especially in the pediatric population with a low short-term risk [1].

MS, formerly a disorder affecting mainly adults, has become a growing problem, also among children. Excessive body weight in early childhood may result in the development of obesity and its future complications [2]. Therefore, appropriate prophylactic management implemented from the earliest age of the patients is important to prevent the development of civilization diseases in the future.

Various diagnostic criteria have been used in the diagnosis of MS. In 2007 the International Diabetes Federation (IDF) developed the criteria for MS in children and adolescents taking into consideration the age brackets. For children aged ≥ 10 years old, MS can be diagnosed with abdominal obesity and the presence of two or more other clinical abnormalities (elevated triglycerides (TG), low *high*-*density lipoprotein* cholesterol (HDL-C) concentration, high blood pressure, increased plasma glucose). Establishing the diagnosis of MS in children below 10 years of age is not recommended by IDF, but the necessity of further diagnostics and observations in case of abnormalities found in this age group is indicated [3]. It should also be emphasized that due to the increasing prevalence of obesity in the pediatric population, it is necessary to determine the risk of diabetes and cardiovascular disease developing in younger children.

## Aim

The general aim of this study was to determine the prevalence of particular MS components in overweight or obese children. Moreover, the effect of risk factors on the incidence of MS was analyzed.

## Material and methods

The study was conducted in a group of 70 overweight and obese children aged 5-18 hospitalized in the Department of Gastroenterology, Allergology and Pediatrics, Polish Mother’s Memorial Hospital – Research Institute in Lodz, Poland in 2013/2014. The patients were divided into three age groups: 5-10 years, 11-16 years and >16 years of age. The medical history of each subject was obtained according to standardized medical files. The analysis included the following data: birth weight, gestation age, breastfeeding (yes/no and duration [months]), as well as family history of hypertension, obesity, diabetes and lipid disorders. All the patients underwent the essential anthropometric measurements: body weight (kg), height (cm), waist circumference (cm). Overweight and obesity were diagnosed on the basis of centile charts for Body Mass Index (BMI) developed by Kułaga and Litwin [4].

The results of arterial blood pressure measurements were interpreted using the centile charts developed by Kułaga and Litwin, taking into account the age, gender and body height centile level. In the group of children below 16 years of age, the blood pressure values ≥95^th^ centile were interpreted as arterial hypertension [5]. In the population of children above 16 years of age, arterial hypertension was diagnosed when the arterial blood pressure values were equal or exceeded 130/85 mmHg, in accordance with the IDF 2007 criteria [3].

The patients underwent a physical examination as well as laboratory investigations, i.e. lipid profile - total cholesterol (TC), HDL-C, low-*density lipoprotein cholesterol (*LDL-C) and TG, fasting glucose level. Insulin resistance was also determined using the Homeostatic Model Assessment of Insulin Resistance (HOMA-IR) index, with the index calculated according to the following formula: fasting insulinemia (μIU/ml) × fasting glycemia (mmol/l)/ 22.5. Insulin resistance was diagnosed when HOMA-IR exceeded 2.67 in boys and 2.22 in girls in the prepubertal period and 5.22 in boys and 3.82 in girls, respectively, in the pubertal period [6].

MS diagnosis was based on the IDF criteria of 2007: visceral fat obesity ≥ 90^th^ percentile and 2 of the other disorders: elevated TG (≥150 mg/dl), reduced HDL-C concentration (HDL-C <40 mg/dl), elevated arterial blood pressure (≥ 95^th^ centile or systolic ≥130 mmHg, diastolic ≥85 mmHg), elevated fasting glycemia (≥100 mg/dl) [3]. The choice of the diagnostic method was determined by the fact that it is simple, easy to use and popular.

In the study group the number of particular risk factors for MS development was analyzed. The following abnormalities were specified as risk factors: visceral obesity, insulin resistance, as well as family history of arterial hypertension, diabetes, obesity and lipid disorders (factors that may determine genetic predisposition) [7]. Moreover, the influence of the duration of pregnancy, birth weight, breastfeeding on MS development was also assessed.

For the purpose of conducting the study, formal written consent of the parents and children aged ≥ 16, as well as the approval of the Polish Mother’s Memorial Hospital – Research Institute Ethics Committee were obtained (90/2015).

## Statistical analysis

For statistical interpretation of the material obtained, the minimum and maximum values were specified and arithmetic means calculated for measurable parameters, together with the calculation of the parameter specifying the internal differentiation of the variables analyzed: standard deviations. For qualitative characteristics, percentages (frequencies) of their particular categories were determined. The significance of the differences between the frequencies of various non-measurable characteristic categories occurring in the groups was assessed using the chi-square test of independence, or the chi-square test of independence with Yates correction, or the Fisher exact test. The Shapiro-Wilk test was used to characterize the distribution of quantitative variables. The significance of the differences in the average values of the variables analyzed in two groups was assessed using the Mann-Whitney test (because the distribution of characteristics deviated from the normal distribution). The risk of developing MS and obesity in relation to the characteristics taken into account in the study was assessed by means of univariate logistic regression. In allthecomparisons,α≤0.05 was adopted as the level of significance. The differences or correlations for which p≤α were regarded as statistically significant. The calculations were carried out using the STATISTICA v.10 software package.

## Results

The study was performed on a group of 70 children (41 girls – 58.6% and 29 boys – 41.4%). The study group comprised 14 children aged 5-10 years; 44 and 12 subjects were included into the age groups 11-16 years and > 16 years, respectively (mean age 12.92; SD 2,9). Distribution of the groups of children in terms of gender and age is presented in Table I.

### Overweight and obesity

Overweight children accounted for 25.7% (n=18) of the study group, whereas obesity was diagnosed in 74.3 % of the children examined (n=52).

### Metabolic syndrome diagnosis

The criteria necessary for MS diagnosis were present in 14 children (20.0%), more frequently in the group aged 11-16 (n=11, 78.6%). In the 5-10-year-old group the features of MS were observed in only one child (7.1%).

### Lipid and carbohydrate disorders, hypertension

In the study populations, mean HDL-C was 46.9 mg/dl (SD 10.6), LDL-C was 99.55 mg/dl (SD 29,7) and TG was 115.3 mg/dl (SD 65.9).

In the group of children with MS, a decrease of HDL-C levels, as well as an increase of TG concentrations were more frequently observed (92.8%; n=13 and 71.4%; n=10) than in the subjects without the MS diagnosis (12.5%; n=7 and 8.9%; n=5) (p<0.05). In the group of subjects with MS arterial hypertension was shown in 71.4% (n=10) cases, while elevated blood serum glucose – was detected in only one child (7.1%). No statistical differences between the groups with and without MS related to arterial hypertension and elevated blood serum glucose were observed (p>0.05). Diabetes mellitus type II was not reported. The frequency of visceral obesity, lipid and carbohydrate disorders, as well as the occurrence of arterial hypertension in the groups studied was shown in Table II.

**Tabela I:** Rozkład badanych w zależności od płci i wieku. Table I. Distribution of subjects in terms of gender and age.

Age (years) *Wiek (lata)*	Girls *Dziewczynki*		Boys *Chłopcy*		Total *Razem*	
	n	%	n	%	n	%
Group 1 (5-10) Grupa 1 (5-10)	10	71.4	4	28.6	14	100.0
Group 2 (11-16) Grupa 2 (11-16)	24	54.5	20	45.5	44	100.0
Group 3 (>16) Grupa 3 (>16)	7	58.3	5	41.7	12	100.0
Total Razem	41	58.6	29	41.4	70	100.0

n − number of subjects n − liczba badanych

### Insulin resistance

Among all the children, insulin resistance was diagnosed in 29 subjects (41.4%) with no differences between the groups of girls and boys (n=17, 41.5% *vs*. n=12, 41.4% respectively) (p>0.05). It was significantly more frequent in the group aged 11-16 (n=25, 56.8%) than in younger children (n=4, 28.6%) (p<0.05). In the group of subjects > 16 years of age insulin resistance was not observed.

### Analysis of the influence of risk factors on MS development

The results of univariate logistic regression indicated that the occurrence of lipid disorders, obesity, hypertension and diabetes in the children’s parents, as well as the duration of pregnancy, birth weight, breastfeeding were not associated with the risk of MS developing in the subjects (p>0.05) (Table III).

The analysis of visceral obesity incidence, insulin resistance in the subjects studied, as well as family history of arterial hypertension, diabetes, obesity and lipid disorders showed that 92.9% of the children had one or more risk factors for MS development, while 71.4% had two or more of them. None of the subjects had all of the factors analyzed (Table IV).

## Discussion

As can be seen from the epidemiological data, the number of overweight and obese children is increasing, which is leading to epidemic proportions around the world [8]. This trend is also observed in the population of Polish children. In a study of 970 children from the Malopolska region, the prevalence of overweight and obesity was noted at 10.2% and 4.2%, respectively [9]. Compared with other Polish studies, the prevalence of MS showed in our study was higher than in the cohort described by Firek-Pędras et al. (14%) [10] but lower than observed by Skowrońska et al. (31%) [11]. This fact probably results from the differences in the age and overweight/obese stages between the studied subjects. According to the current recommendations, MS is not diagnosed in patients below 10 years of age but taking into consideration the growing problem of obesity in the pediatric population we also included younger children in our observations. The differences in the MS rate in studies also depends on the varying diagnostic criteria used by the authors. In our study MS diagnosis was based on the IDF criteria of 2007, while in the population of US adolescents where the ATP-III criteria were used 9.2% had MS (31.2% of overweight/obese adolescents) [12]. On the other hand, Cook et al., who based their study on less restrictive criteria than Ferranti, noted MS in 4.2% of the adolescents (28.7% of overweight adolescents) [13]. IDF criteria are less restrictive than those mentioned above proposed by Cook and de Ferranti in terms of a decrease of HDL and increase of TG levels, as well as waist circumference, however they suggest the lower glucose fasting cutpoint of 100 mg/dl (in other criteria ≥110 mg/dl). The lack of universal criteria for MS recognition in the population of children, especially in subjects below 10 years of age, as well as different eating habits in particular countries, make it difficult to compare the results of the authors.

**Tabela II:** Częstość otyłości trzewnej, zaburzeń gospodarki lipidowej i węglowodanowej oraz nadciśnienia tętniczego u badanych. Table II. Frequency of visceral obesity, lipid and carbohydrate disorders as well as arterial hypertension in the studied subjects.

		MS+(n=14)		MS-(n=56)		Total *Ogółem (n=70)*
		Overweight *Nadwaga*	Obesity *Otyłość*	Overweight *Nadwaga*	Obesity *Otyłość*	
Visceral obesity *Otyłość trzewna*	n	3	11	11	39	64
	%	21.4	78.6	19.6	69.6	91.4
Decreased HDL-C levels *Obniżone stężenie HDL*	n	3	10	1	6	20
	%	21.4	71.4	1.8	10.7	28.6
Increased TG levels *Podwyższone stężenie TG*	n	3	7	3	2	15
	%	21.4	50.0	5.3	3.6	21.4
Increased glucose levels Podwyższone stężenie glukozy	n	-	1	-	-	1
	%	-	7.1	-	-	1.4
Arterial hypertension *Nadciśnienie tętnicze*	n	1	9	8	20	36
	%	7.1	64.3	14.3	35.7	51.4

n − number of subjects, n − liczba badanych; MS − Metabolic syndrome, MS − zespół metaboliczny Statistically significant differences between two groups, i.e. MS+ and MS- regarding decreased HDL-C and increased TG levels were observed (p<0.05). *Istotności statystyczne pomiędzy badanymi grupami MS+ oraz MS- zostały stwierdzone w zakresie parametrów: obniżonego stężenia HDL-C oraz podwyższonego stężenia TG (p<0,05)*.

**Tabela III:** Ryzyko wystąpienia zespołu metabolicznego w zależności od analizowanych cech − wyniki jednoczynnikowej regresji logistycznej. Table III. Risk of metabolic syndrome related to the characteristics analyzed – results of univariate logistic regression.

Variable *Zmienna*		OR	95% CI	p
Breastfeeding *Karmienie naturalne*	Yes *[Tak]*	0.61	0.16-2.38	0.47
	No *[Nie]*	1.00	Ref.	
Breastfeeding (months) *Karmienie naturalne (miesiące)*	-	0.99	0.90-1.08	0.76
Familial history of hypertension *Występowanie w rodzinie nadciśnienia tętniczego*	Yes	1.08	0.32-3.60	0.90
	No	1.00	Ref.	
Obese parents *Otyłość u rodziców*	Yes	0.55	0.11-2.87	0.47
	No	1.00	Ref.	
Diabetic parents *Cukrzyca u rodziców*	Yes	1.46	0.43-4.93	0.53
	No	1.00	Ref.	
Familial history of lipid disorders *Zaburzenia lipidowe w rodzinie*	Yes	2.27	0.48-10.81	0.29
	No	1.00	Ref.	
Birth weight (kg) *Masa urodzeniowa (kg)*	-	0.75	0.24-2.31	0.60
Gestational age (week) *Wiek ciążowy (hbd*)	-	0.85	0.65-1.11	0.23

**Tabela IV:** Liczba czynników ryzyka wystąpienia zespołu metabolicznego w grupie badanych. Table IV. Number of risk factors for metabolic syndrome development in the study group.

Number of risk factors *Liczba czynników ryzyka*	n	%
0	5	7.1
1	15	21.4
2	19	27.1
3	13	18.6
4	11	15.7
5	7	10.0
6	0	-

Risk factors for metabolic syndrome development: visceral obesity, insulin resistance, familial history of: arterial hypertation, diabetes, obesity, lipid disorders. *Czynniki ryzyka rozwoju zespołu metabolicznego: otyłość trzewna, insulinooporność, występowanie w rodzinie: nadciśnienia tętniczego, cukrzycy, otyłości, zaburzeń lipidowych*.

A sedentary lifestyle, low or no physical activity, as well as a diet rich in high fat and high calorie food products lead to increasing the incidence of visceral obesity. Obesity entails a cascade of other metabolic disorders, such as abnormalities of carbohydrate and lipid metabolism, or increase of arterial blood pressure [14]. Firek-Pędras et al. demonstrated that hypertension occurs in 18% of obese children, whereas lipid disorders were confirmed in 50% of the children with body weight abnormalities [10]. In a study of 325 children from the Lodz region, the risk of the onset of elevated triglyceride concentration was 16-fold higher in children with central obesity [15]. In our study we also observed a correlation of excess body weight with lipid disorders in the examined group of children. The most prevalent MS component, followed by abdominal obesity, was decreased HDL-C concentration. According to some investigators, it is an alarming trend. Ozer et al. showed that low HDL-C levels are the most significant risk factor for early atherosclerosis in children with MS [16]. In our study diabetes mellitus type II was not reported, however insulin resistance (IR), which is a very important element in the diagnostics of metabolic disorders, was observed in 41.4% of subjects. The above-mentioned IR refers to glucose homeostasis disorders, the essence of which is the reduced sensitivity of tissues to the effect of insulin, despite its normal or elevated concentration in blood serum. IR as an etiopathogenetic factor for MS and associated compensative hyperinsulinemia with impaired glucose tolerance or overt diabetes seem to be independent risk factors for cardiovascular diseases, also affecting the pathogenesis of other elements contributing to the risk of circulatory system pathologies, such as arterial hypertension, dyslipidemia or hemostasis disorders [17].

Our study showed that in the group of older children (>10 years old) all the components necessary for MS diagnosis were more frequently observed than in the population of younger subjects. In our opinion it results from a longer exposure to risk factors, such as bad trends in human nutrition (overeating, high calorie food) and minimum physical activity that leads to the promotion of weight gain and metabolic disorders. However, the excessive number of adipose cells developed as early as in fetal life, or during the initial period of extrauterine life, has a considerable impact on the occurrence of obesity in adults. A study conducted by Danish researchers confirmed that subjects with increased BMI in childhood more frequently suffered from cardiovascular complications in the future [18]. Therefore, healthy dietary habits and physical activity during pregnancy, as well as a healthy balanced diet of the offspring are extremely important [19, 20].

Predispositions to overweight and obesity, and in consequence to the development of MS, are influenced by how neonates and infants are fed. In their previous studies the authors indicated that not only does mother’s milk have nutritional, immune and trophic properties for the growing and maturing organism, but it may also protect them from the development of metabolic disorders in adulthood [21, 22]. In our study we did not observe a statistically significant influence of breastfeeding in the past and the prevalence of MS in the groups analyzed. However, the group of breastfed children included both exclusively breastfeed subjects as well as mixed-fed subjects. Holmes et al. indicated that the combination of breast milk and formula feeding, as well as formula feeding as compared with breastfeeding exclusively were associated with an increased risk of overweight and obesity between the ages of 2 and 6 years [23]. Moreover, similarly to the research performed by Yakubov et al., the influence of the duration of breastfeeding on the reduction of the risk of MS occurring was not confirmed in our study groups [24].

Previous studies showed that low birth weight is one of the factors leading to obesity, diabetes, increased leptin levels, as well as MS development in the future [25, 26]. In our analysis no significant differences indicating the effect of birth weight and gestation age on MS diagnosis were observed. However, the study was performed only on the group of children with overweight and obesity without a control group with normal BMI, which may influence the results. Moreover, in our observation, familial history of arterial hypertension, lipid disorders and diabetes were not observed significantly often in the group of children with MS in comparison to other subjects. In our opinion this results from the strong influence of environmental factors, such as high calorie nutrition and the lack of physical activity from early childhood, which contribute to obesity and MS development in a greater way than perinatal and genetic factors. However, it may also be the effect of too small a number of children included in the groups that were analyzed, which is another limitation of this study.

## Conclusions

The high percentage of lipid disorders in the group of children with MS may have a prognostic significance, especially concerning the risk of developing atherosclerosis.

The occurrence of one or more MS risk factors in almost all of the overweight and obese children indicates the increased future risk for cardiovascular diseases in the studied group.

Early detection of the risk of MS will enable the implementation of appropriate therapeutic interventions resulting in the inhibition of excessive body mass increases, reduce the risk for future cardiovascular incidents, as well as other complications of overweight and obesity.

Further multifactorial analyses performed on representative groups are necessary to specify the risk factors for MS development in children and adolescents.
